# Clinical Ultrasound Is Safe and Highly Specific for Acute Appendicitis in Moderate to High Pre-test Probability Patients

**DOI:** 10.5811/westjem.2018.1.36891

**Published:** 2018-03-13

**Authors:** Daniel Corson-Knowles, Frances M. Russell

**Affiliations:** Indiana University School of Medicine, Department of Emergency Medicine, Indianapolis, Indiana

## Abstract

**Introduction:**

Clinical ultrasound (CUS) is highly specific for the diagnosis of acute appendicitis but is operator-dependent. The goal of this study was to determine if a heterogeneous group of emergency physicians (EP) could diagnose acute appendicitis on CUS in patients with a moderate to high pre-test probability.

**Methods:**

This was a prospective, observational study of a convenience sample of adult and pediatric patients with suspected appendicitis. Sonographers received a structured, 20-minute CUS training on appendicitis prior to patient enrollment. The presence of a dilated (>6 mm diameter), non-compressible, blind-ending tubular structure was considered a positive study. Non-visualization or indeterminate studies were considered negative. We collected pre-test probability of acute appendicitis based on a 10-point visual analog scale (moderate to high was defined as >3), and confidence in CUS interpretation. The primary objective was measured by comparing CUS findings to surgical pathology and one week follow-up.

**Results:**

We enrolled 105 patients; 76 had moderate to high pre-test probability. Of these, 24 were children. The rate of appendicitis was 36.8% in those with moderate to high pre-test probability. CUS were recorded by 33 different EPs. The sensitivity, specificity, and positive and negative likelihood ratios of EP-performed CUS in patients with moderate to high pre-test probability were 42.8% (95% confidence interval [CI] [25–62.5%]), 97.9% (95% CI [87.5–99.8%]), 20.7 (95% CI [2.8–149.9]) and 0.58 (95% CI [0.42–0.8]), respectively. The 16 false negative scans were all interpreted as indeterminate. There was one false positive CUS diagnosis; however, the sonographer reported low confidence of 2/10.

**Conclusion:**

A heterogeneous group of EP sonographers can safely identify acute appendicitis with high specificity in patients with moderate to high pre-test probability. This data adds support for surgical consultation without further imaging beyond CUS in the appropriate clinical setting.

## INTRODUCTION

Acute appendicitis is inflammation of the appendix that can lead to perforation, abscess, other serious infections and death. Over 280,000 appendectomies are performed in the United States annually.^1^ Although widespread availability of computed tomography (CT) has allowed more accurate diagnosis of acute appendicitis before reaching the operating room, this has come at the price of increased radiation exposure, increased cost and longer emergency department (ED) lengths of stay.^2^–^4^

Due to these risks, it is common to perform ultrasound examinations as the initial imaging modality in children to diagnose acute appendicitis.^5^ Nonetheless, ultrasonography for appendicitis is not available in many EDs, and in most departments the availability of diagnostic ultrasonography is limited by the time of day.^6^,^7^ Even when available, the accuracy of formal radiology ultrasound may be much lower in community practice than in academic centers where it is commonly studied.^8^

Previous studies have demonstrated excellent specificity of point-of-care or clinical ultrasound (CUS) for acute appendicitis among small cohorts of highly trained sonographers,^9^–^15^ and incorporation of clinical risk-stratification with sonography has been shown to safely enhance diagnostic accuracy in a variety of settings.^16^–^20^ However, the accuracy of ultrasound is highly dependent on the skills of the operator. This may be a barrier to implementation of CUS for appendicitis in new settings. The goal of this study was to determine if a heterogeneous group of emergency physicians (EP) could diagnose acute appendicitis on CUS. We hypothesized that EP sonographers could diagnose acute appendicitis with high specificity using a combination of clinical risk assessment, CUS, and self-assessment of image acquisition and interpretation.

## METHODS Study Design

This was a prospective observational study on a convenience sample of adult and pediatric patients presenting to the ED with signs and symptoms concerning for acute appendicitis. Patients were enrolled from three large urban academic EDs between July 2014 and September 2016. The study sites consisted of two adult centers with a combined annual census of approximately 205,000, and one pediatric center with an annual volume >40,000 patient visits. The study protocol was approved by the institutional review board.

We included any patient with suspected acute appendicitis who underwent a diagnostic EP-performed CUS. Children and pregnant women were included. We excluded patients if CUS images were obtained after formal radiology imaging, or if data collection forms had missing information (including patient demographic information, pre-test or post-test probability, or interpretation).

### Study Protocol

CUS was performed at the discretion of the treating clinician after history and physical examination. Prior to CUS, the treating physician recorded pre-test probability of appendicitis on a 10-point visual analog scale (VAS) using clinical gestalt. Following ultrasound, the sonographer filled out a standardized data collection form including the ultrasound diagnosis (appendicitis, indeterminate, or no appendicitis) and confidence in ultrasound interpretation on a 10-point VAS.

Sonographers included emergency medicine residents, ultrasound fellows, and board-certified emergency medicine faculty. All sonographers underwent a structured, 20-minute CUS training on appendicitis including didactics and hands-on scanning of one live model. CUS was performed after parenteral analgesics using a linear 5–10 MHz probe (Zonare ZS3 or Z.One Pro, Mindray Zonare, Mountain View, CA). The patient was in a supine position with hips flexed to relax the abdominal musculature. Graded compression was applied over the patient’s maximal site of pain in the right lower quadrant of the abdomen. The presence of a dilated (>6 mm diameter), non-compressible, blind-ending (in long axis) tubular structure was considered a positive study. Secondary signs of appendicitis were not assessed. Non-visualization or indeterminate studies were considered negative.

### Outcome

The primary outcome was the diagnostic accuracy of CUS for acute appendicitis in patients with moderate and high pre-test probability. We used unstructured clinical assessment on VAS to determine pre-test probability, as clinical judgment has been shown to outperform clinical decision tools such as the Alvarado score.^21^ Pre-test probability of appendicitis was grouped into categories of low (1–3), moderate (4–6), and high (7–10). The criterion standard for diagnosis was surgical pathology results for those patients who went to the operating room, and chart review at hospital discharge and one week post-index ED visit for patients who did not go to the operating room. Local and statewide electronic medical records (EMR) were reviewed for repeat ED visits or hospitalizations for missed cases. We defined a missed case of acute appendicitis as a discharge diagnosis or surgical pathology diagnosis of acute appendicitis after the index visit.

### Data Analysis

The expected rate of appendicitis was 35%.^11^–^13^ We expected specificity to be 85%, based on prior studies demonstrating a specificity ranging from 71 to 91%.^17^ A sample size of 75 patients with moderate to high pre-test probability of appendicitis was planned to demonstrate specificity within 10% of the expected value. This calculation assumes a power of 0.80 and alpha of 0.05. We calculated sensitivity, specificity, and positive and negative likelihood ratios with 95% confidence intervals (CI). Twenty percent of studies were randomly selected for blinded review by a fellowship-trained expert to calculate observed agreement and inter-rater reliability between EP sonographers’ interpretations using Cohen’s unweighted kappa. The expert reviewer was blinded to sonographer identity, sonographer interpretations, and clinical data.

## RESULTS

During the study period 122 patients underwent CUS. Seventeen studies were excluded for missing data on the data collection form, including missing or incorrect patient medical record numbers, missing sonographer interpretation or pre-test probability. Of the remaining 105 patients, 76 (72%) had moderate or high pre-test probability (see Figure). Of these 76 patients, 28 (36.8%) had acute appendicitis (Table 1). There were 27 children, of whom 24 had moderate or high pre-test probability for appendicitis. Two pregnant women underwent CUS and both had a low pre-test probability. At one-week, EMR follow-up there were no missed cases of acute appendicitis.

The sensitivity and specificity of EP-performed CUS in patients with moderate to high pre-test probability were 42.8% (95% CI [25–62.5%]) and 97.9% (95% CI [87.5–99.8%]) (Table 2). The positive and negative likelihood ratios were 20.6 (95% CI [2.8–149.9]) and 0.58 (95% CI [0.42–0.8]). In 31 studies sonographers reported high confidence in image acquisition and interpretation (6 or higher on a 1–10 VAS). Of these studies, the sensitivity and specificity improved to 80% and 100%, respectively. The 16 false negative scans all were interpreted as indeterminate; for all 16, appendicitis was confirmed by CT at the index visit. There was one false positive ultrasound. For this study the sonographer reported low confidence in image interpretation (2 out of 10). This patient had a CT that demonstrated an obstructing ureteral stone at the right ureterovesical junction. Two patients proceeded directly to the operating room for appendectomy based on a positive CUS with no further imaging.

Thirty-three different sonographers performed CUS with a range of 1–13 scans per sonographer. Residents performed 40 (52.6%) of the CUS and identified five (41.7%) of the true positives. Inter-rater reliability was high, with 100% agreement and kappa = 1 (95% CI [1–1]).

## DISCUSSION

Our study demonstrates that a heterogeneous group of EPs can diagnose acute appendicitis by CUS with high specificity in the appropriate clinical context. In our study, most sonographers had performed an average of 100 prior CUS examinations. Pre-enrollment training was limited to 20 minutes of didactics and hands-on training with a healthy model.

Recent research has shown diagnostic accuracy can be improved by combining clinical assessment with sonography.^16^–^20^ Therefore, we identified patients with moderate to high pre-test probability. Since ultrasound accuracy is highly dependent on the skill of the sonographer, we also collected data on sonographer confidence. Higher confidence in the ultrasound diagnosis yielded improved sensitivity and specificity of the results. This suggests that confidence in image acquisition and interpretation is an important predictor of diagnostic accuracy for CUS.

These results are consistent with prior studies demonstrating high specificity and moderate sensitivity of EP-performed CUS for acute appendicitis. In a meta-analysis of 21 studies, Fields et al. showed high specificity of 92% and relatively high sensitivity of 80% for CUS by EPs.^15^ Our sensitivity is lower than that reported by Fields et al. because the authors excluded non-diagnostic studies from analysis. These results further support the use of CUS as a first-line imaging modality in patients with suspected appendicitis. CUS has potential advantages as compared to CT with reduced time to diagnosis, reduced costs, reduced radiation and contrast dye exposure, and shorter ED stays.^2^,^4^

## LIMITATIONS

The generalizability of this study is limited by the use of a convenience sample design and small number of subjects. There may be spectrum bias based on the inclusion criteria. EP sonographers were not blinded to patient history or physical exam, which could impact real-time interpretation of the images. However, this reflects pragmatic use of CUS in EDs during the early adoption period. The rates of acute appendicitis in this cohort are consistent with prior studies, suggesting that physicians used CUS in a group of patients similar to those seen in routine clinical practice. Follow-up was limited to one-week chart review, but it is unlikely any patients were missed due to use of a statewide-linked EMR. Although highly specific, the sensitivity of 42.8% does not support the use of CUS to rule out acute appendicitis in moderate to high pre-test probability patients.

## CONCLUSION

A heterogeneous group of EPs can safely identify acute appendicitis on CUS with high specificity and a positive likelihood ratio of 20. Clinical risk stratification and appraisal of image quality and interpretation may improve diagnostic accuracy. Surgical consultation without further imaging beyond CUS may be supported in the appropriate clinical setting. This data does not support the use of CUS to rule out appendicitis when there is persistent clinical concern.

## Figures and Tables

**Figure:**
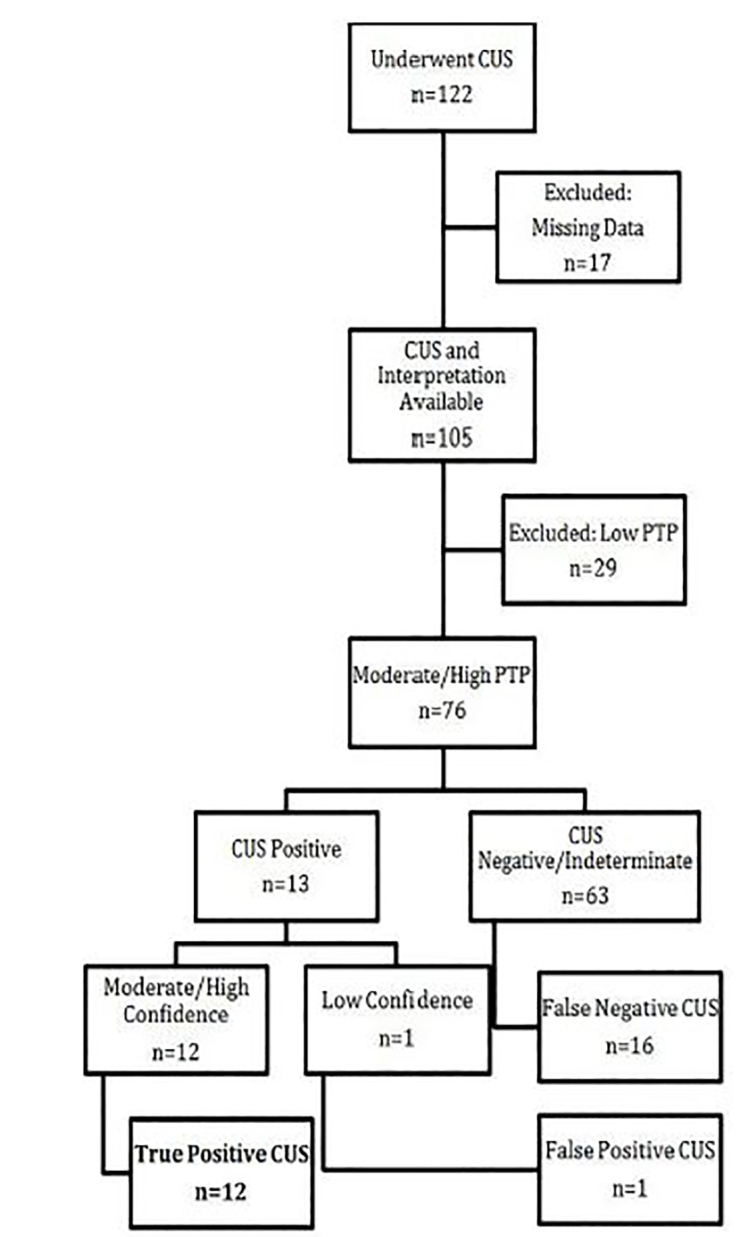
Flow chart of patients with moderate to high risk appendicitis and use of clinical ultrasound. Among patients with moderate to high risk of appendicitis, clinical ultrasound identified 12/28 cases of acute appendicitis. Among positive CUS scans, all tests with high sonographer confidence were true positives. *CUS*, clinical ultrasound; *PTP*, pre-test probability

**Table 1:** Patient characteristics with moderate to high pre-test probability.

	Appendicitis (n=28)	No appendicitis (n=48)
Age, mean	23.9 ±13.1	28.7 ±16.1
Age <18	12	12
Sex (M)	69.6%	59.5%
BMI	24.5 ±6.6	26.3 ±4.7
Symptom duration (d)	1.1 ±1.1	1.5 ±1.6
Fever	32.1%	27.1%
Vomiting	50.0%	41.7%
Rebound	46.4%	27.1%
Migration	82.1%	27.1%
Anorexia	78.6%	56.3%
White blood cells	12.9 ±3.8	10.7 ±5
Alvarado score	4.6 ±1.4	2.2 ±1.3
Formal radiology imaging	90%	90%

**Table 2:** Sensitivity and specificity by level of training.

	Faculty	Fellow	Resident	Total
N	25	11	40	76
Sensitivity	50%	75%	31%	43%
Specificity	100%	100%	96%	98%
